# Exacerbations and crises in generalized myasthenia gravis: predictors and associated economic burden in the United States

**DOI:** 10.3389/fneur.2026.1919726

**Published:** 2026-10-19

**Authors:** Kavita Grover, Martin Cloutier, Geoffroy Coteur, Porpong Boonmak, Antoine C. El Khoury, Maryia Zhdanava, Anabelle Tardif-Samson, Yuxi Wang, Kavita Gandhi

**Affiliations:** 1 Henry Ford Health System, Detroit, MI, United States; 2 Wayne State University School of Medicine, Detroit, MI, United States; 3 Analysis Group, Inc., Montreal, QC, Canada; 4 Johnson and Johnson, Horsham, PA, United States; 5 iPATH Solutions, BV, Wemmel, Belgium; 6 Johnson and Johnson, Raritan, NJ, United States

**Keywords:** exacerbations, generalized myasthenia gravis, healthcare resource utilization and cost, myasthenic crisis, predictors

## Abstract

**Introduction:**

The management of exacerbations and crises (clinical events) in generalized myasthenia gravis (gMG) patients is resource-intensive. Identifying characteristics associated with increased risk for these events may help optimize allocation of preventative strategies. This study identified baseline predictors of a first clinical event following diagnosis and assessed the burden of these events among patients with gMG in the United States.

**Methods:**

A newly diagnosed cohort of adults with gMG were identified from Komodo Research Database (01/2017–09/2023) with an index date at the first gMG diagnosis made by a neurologist. Baseline demographics, comorbidities, gMG-related treatments, healthcare resource utilization (HRU) and costs were described; patients were followed until first clinical event or censoring at end of follow-up. A multivariable Cox proportional hazards model assessed baseline predictors of the first clinical event. Among patients with ≥12 months of follow-up, cohorts with and without a clinical event during follow-up were created; index date was the first clinical event or a random date, respectively. Cohorts were re-weighted using entropy balancing. HRU and costs during 12 months post-index were compared.

**Results:**

Among 6,195 patients with gMG (mean age: 61.1 years; female: 49.1%), higher event risk was associated with use of immunoglobulin [hazard ratio (HR) = 1.54], baseline dysarthria (HR = 1.45), general and other muscle-specific weaknesses (HR = 1.38), use of non-steroidal immunosuppressants (HR = 1.37), dysphagia (HR = 1.28), weight loss (HR=1.27), dyspnea (HR = 1.23), ptosis (HR = 1.20), absence of other autoimmune disorders (HR=1.18), and male sex (HR = 1.15; all *p* < 0.05). In the cohort, 2,657 had a clinical event, while 3,092 did not; all had ≥12 months of follow-up after index. After re-weighting, patients with a clinical event had higher number of inpatient admissions [rate ratio (RR) = 6.02], emergency visits (RR = 1.91), and outpatient visits (RR = 1.58; all *p* < 0.05) in the 12 months post-index. Mean total healthcare costs were $70,792 higher among patients with a clinical event ($95,821 vs. $25,029, *p* < 0.001), driven by immunoglobulin and inpatient costs.

**Discussion:**

Disease severity and immunosuppressive therapy use were associated with increased risk of exacerbations and crises, which imposed substantial HRU and cost burden. Findings may help identify candidates who could benefit from earlier or more advanced therapies to prevent acute clinical events.

## 1 Introduction

Myasthenia gravis (MG) is a rare autoimmune disorder with an estimated prevalence of 37 cases per 100,000 individuals in the United States (US) (1). MG is characterized by fluctuating muscle weakness and fatigue (2). Generalized MG (gMG) involves multiple muscle groups, including limb, axial, and bulbar muscles, and can present with difficulty chewing, talking, swallowing, or breathing (3, 4).

Within any given year, approximately one third of patients with gMG experience myasthenic exacerbations (MEs), which are periods when muscle weakness worsens beyond the patient's usual symptoms level (5). These MEs involve muscles other than the ocular muscles, but do not necessarily result in respiratory failure. MEs signal a need for elevated clinical attention and therapy adjustments to help control the elevated symptoms and often require an emergency department (ED) visit or inpatient admission, which can last 6–9 days on average (6–8). A myasthenic crisis (MC) is a more severe, life-threatening escalation of muscle weakness that leads to respiratory failure, necessitating intensive care unit admission with assisted ventilation, usually requiring an inpatient stay period of several weeks, often in the range of 2–4 weeks (7, 9–11). Approximately 20% of patients with gMG are estimated to experience MC during a period of 3 years or longer (9, 12–14). Patients who experience MEs and MCs may need rescue medication (e.g., plasma exchange or immunoglobulin) (15) and, for MCs, may additionally require supplemental oxygen and intubation. To regain muscle function after MEs and MCs, patients may need rehabilitation therapy (e.g., physiotherapy, balance training, respiratory training) (16).

Preliminary evidence suggests that several factors can contribute to the risk of experiencing MEs and MCs in patients with gMG, including sex, disease severity at the time of gMG diagnosis, and reduction in prednisone dose (17). However, prior analyses were restricted by small sample sizes and limited patient settings, or were conducted in patients with prevalent gMG with a specific serotype, potentially missing early ME and MC events and limiting generalizability to the broader gMG population (6, 17, 18). While the economic burden of gMG has also been previously estimated, there remains limited evidence regarding the specific economic and clinical burden associated with MEs and MCs. To address this gap, this study aimed to characterize, in a broad and representative sample of newly diagnosed patients with gMG in the US, which patients are at greatest risk of a gMG-related clinical event and the clinical and economic consequences associated with these events. Accordingly, two distinct but complementary analyses were conducted: the first identified predictors of gMG-related clinical events (i.e., MEs and MCs), and the second estimated the incremental healthcare resource utilization (HRU) and costs associated with these events.

## 2 Methods

### 2.1 Data source

This study used administrative claims data from the Komodo Research Database (KRD+) covering the period from January 1, 2017, through September 30, 2023. KRD+ aggregates medical and pharmacy claims directly from more than 150 payers and represents a population of over 160 million patients across the US. Available data include patient demographics (sex, year of birth, race/ethnicity), health plan eligibility dates, diagnoses and procedures, service dates, provider and payer types, and costs for medical and pharmacy claims. Costs associated with pharmacy and medical claims are imputed and based on the maximum amount an insurer was allowed for the service. The database incorporates claims from commercial, Medicare Advantage, and Medicaid populations, thereby capturing a large and diverse cohort of patients. All data in KRD+ are de-identified and compliant with the Health Insurance Portability and Accountability Act; therefore, institutional review board approval was not required.

### 2.2 Study design

The study had a retrospective cohort design.

#### 2.2.1 Predictors of a clinical event

Predictors of clinical events were assessed among all newly diagnosed patients with gMG (*gMG* cohort). The index date was defined as the date of the first evidence of gMG, as defined in the Selection Criteria below. The baseline period included 12 months before the index date. Patients were also required to have ≥12 months of continuous insurance eligibility after the index date. The follow-up period spanned from the index date until the earliest among (i) the date of the first clinical event following the first evidence of gMG, (ii) the end of continuous insurance eligibility, or (iii) the end of data availability.

#### 2.2.2 Burden of a clinical event

To assess the incremental economic burden of a clinical event (i.e., comparing HRU and costs between those with and without a clinical event), the following cohorts were identified among patients with gMG: (i) patients with gMG who experienced a clinical event (i.e., MC or ME; *clinical event* cohort) during follow-up, and (ii) patients with gMG who did not experience a clinical event (*no clinical event* cohort) during follow-up. In this analysis, the index date for the *clinical event* cohort was defined as the date of the first clinical event after gMG evidence, and for the *no clinical event* cohort, it was defined as a random date during the period after gMG evidence. The baseline period included the 12 months before the index date, and the follow-up period started on the index date and lasted for 12 months.

### 2.3 Selection criteria

#### 2.3.1 Predictors of a clinical event

Adult patients with newly diagnosed gMG were eligible for study inclusion. Given that there is no specific diagnostic code for gMG, evidence of gMG was established using a combination of diagnosis codes for MG, care setting, and physician specialty [adapted from Phillips et al. (19) and Zhdanava et al. (20)].

Specifically, patients were considered to have evidence of gMG if they had (i) ≥1 claim with a diagnosis for MG [International Classification of Diseases, 10th Revision, Clinical Modification (ICD-10-CM): G70.00, G70.01] in a primary position in an inpatient setting or ≥2 claims with diagnosis for MG in any position and in any setting ≥1 month apart, and (ii) ≥1 diagnosis of MG made by a neurologist; this requirement excluded patients managed exclusively by an ophthalmologist. The first diagnosis of MG by a neurologist served as the date of first claims-based evidence of gMG.

To ensure that only patients with newly diagnosed gMG were selected, patients were required to have ≥12 months of continuous insurance eligibility before the first MG diagnosis. Patients were further required to be ≥18 years old on the date of first evidence of gMG, have no claim for congenital MG (ICD-10-CM code: G70.2) within 12 months before the first evidence of gMG, and have ≥12 months of continuous insurance eligibility after it.

#### 2.3.2 Burden of a clinical event

Patients from the *gMG* cohort were further included in the *clinical event* cohort if they had ≥1 clinical event following the first evidence of gMG (i.e., a new ME or MC encounter following a neurologist-confirmed gMG diagnosis). An ME was defined as an ICD-10-CM diagnostic code G70.01 (Myasthenia gravis *with* acute exacerbation) in any position or setting, or G70.00 (Myasthenia gravis *without* acute exacerbation) diagnostic code in a primary position and in an inpatient or ED setting (20, 21). An MC was defined as an inpatient or intensive care unit admission with an MG diagnostic code on the same claim with a procedure for intubation, tracheostomy or mechanical ventilation (20, 21). Encounters satisfying both definitions were classified as an MC. Patients were included in the *no clinical event* cohort if they had no evidence of a clinical event, as defined above, following the first evidence of gMG.

The date of first clinical event defined the index date for the *clinical event* cohort, and a random date with a medical visit after the first evidence of gMG defined the index date for the *no clinical event* cohort. Patients in both cohorts were additionally required to have 12 months of continuous insurance eligibility after the index date. This 12-month period also defined the follow-up period.

### 2.4 Study outcomes

#### 2.4.1 Predictors of a clinical event

The first clinical event following the first gMG diagnosis was identified based on the definitions of ME and MC above.

#### 2.4.2 Burden of a clinical event

All-cause and gMG-related HRU and healthcare costs were reported per patient per year (PPPY) during the follow-up period. Total healthcare costs were the sum of pharmacy and medical costs. All-cause pharmacy costs included all pharmacy claims and the following gMG-related classes of therapies incurred under medical costs: immunoglobulin, rituximab, complement-5 inhibitor, and neonatal Fc receptor blocker. All-cause medical costs included all medical claims, excluding costs for the classes of gMG-related therapies above, and were reported by care setting (i.e., inpatient, ED, outpatient, other).

Medical costs related to gMG were based on medical claims with an MG diagnosis in any position; gMG-related pharmacy costs were based on claims for gMG-related medications.

Healthcare costs were adjusted for inflation to 2023 US dollars using the US Consumer Price Index.

### 2.5 Statistical analysis

#### 2.5.1 Predictors of a clinical event

Among the *gMG* cohort, a multivariable Cox proportional hazards model, including demographics, comorbidities, gMG characteristics, including gMG symptoms and number of months from first MG encounter to index date, gMG-related treatment use, and HRU, was used to identify the association of each baseline characteristic while keeping every other variable constant. In the absence of a formal disease severity classification (e.g., MGFA Severity Classifications), baseline gMG symptoms were included as proxies for disease severity. Associations between each baseline characteristic and the first clinical event were reported as hazard ratios (HRs), and characteristics with the *p*-value < 0.05 were considered predictors of experiencing a clinical event. Patients were followed until the first occurrence of a clinical event (event), end of follow-up (censored), or study period (censored).

#### 2.5.2 Burden of a clinical event

Entropy balancing was used to re-weight baseline characteristics of the *no clinical event* cohort to match with those in the *clinical event* cohort. The following characteristics were re-weighted: age, sex, race/ethnicity, US geographic region at index, payer (i.e., commercial, Medicare Advantage, Medicaid, unknown), year of the index date, time from first evidence of gMG to index date, and the presence of MG diagnosis during the baseline period. The balance of baseline characteristics after re-weighting was assessed using standardized differences (< 10% was considered well balanced) (22).

HRU and healthcare costs in the follow-up period were compared between the *clinical event* and *no clinical event* cohorts. Count variables were compared as rate ratios using Poisson regression models. Annual costs during the follow-up period were compared as mean cost difference (MCD) using ordinary least squares regressions. For both count and cost variables, a non-parametric bootstrap procedure with 500 replications was used to produce 95% confidence intervals and *p*-values.

Means, standard deviations, and medians were used to describe continuous variables, and frequencies and proportions to describe categorical variables. All analyses were conducted using SAS^®^ Enterprise Guide^®^ version 8.3 (SAS Institute, Cary, NC, US).

## 3 Results

### 3.1 Predictors of a clinical event

Of the 6,195 patients in the *gMG* cohort, the mean age was 61.1 years, and 49.1% were female. Most patients were White (63.1%), resided in the South (35.5%), and were commercially insured (53.4%). The most common physical comorbidities during the baseline period were hypertension (59.3%), obesity (34.8%), and diabetes (26.1%; Table 1).

**Table 1:** Patient demographic and clinical characteristics during the baseline period.

Mean ±SD [median] or *n* (%)	gMG *N* = 6,195	Clinical event *N* = 2,657	No clinical event *N* = 3,092	Std. diff^a^ %
Age at index date (years)	61.1 ± 15.4 [62.6]	61.4 ± 15.6 [62.7]	61.4 ± 15.4 [63.4]	0.0
Female	3,040 (49.1)	1,262 (47.5)	1,469 (47.5)	0.0
Race/ethnicity				
White	3,908 (63.1)	1,700 (64.0)	1,978 (64.0)	0.0
Hispanic or Latino	489 (7.9)	210 (7.9)	244 (7.9)	0.0
Black or African American	440 (7.1)	199 (7.5)	232 (7.5)	0.0
Asian or Pacific Islander	194 (3.1)	77 (2.9)	90 (2.9)	0.0
Other or unknown	156 (2.5)	61 (2.3)	71 (2.3)	0.0
Unknown	1,008 (16.3)	410 (15.4)	477 (15.4)	0.0
Geographic region				
South	2,199 (35.5)	991 (37.3)	1,153 (37.3)	0.0
Northeast	1,730 (27.9)	697 (26.2)	811 (26.2)	0.0
Midwest	1,359 (21.9)	544 (20.5)	633 (20.5)	0.0
West	907 (14.6)	425 (16.0)	495 (16.0)	0.0
Payer at index date				
Commercial	3,307 (53.4)	1,391 (52.4)	1,619 (52.4)	0.0
Medicare advantage	2,537 (41.0)	1,112 (41.9)	1,294 (41.9)	0.0
Medicaid	350 (5.6)	154 (5.8)	179 (5.8)	0.0
Unknown	1 (0.0)	0 (0.0)	0 (0.0)	0.1
Year of index date				
2018	1,004 (16.2)	344 (12.9)	400 (12.9)	0.0
2019	1,236 (20.0)	493 (18.6)	574 (18.6)	0.0
2020	1,249 (20.2)	541 (20.4)	630 (20.4)	0.0
2021	1,447 (23.4)	688 (25.9)	801 (25.9)	0.0
2022	1,259 (20.3)	591 (22.2)	688 (22.2)	0.0
Common physical comorbidities				
Hypertension	3,675 (59.3)	1,647 (62.0)	1,815 (58.7)	6.7
Obesity	2,155 (34.8)	984 (37.0)	1,055 (34.1)	6.1
Diabetes	1,616 (26.1)	737 (27.7)	797 (25.8)	4.5
Common DSM-5 conditions				
Sleep-wake disorders	1,618 (26.1)	785 (29.5)	790 (25.5)	9.0
Anxiety disorders	1,395 (22.5)	682 (25.7)	617 (20.0)	13.6^*^
Depressive disorders	1,063 (17.2)	518 (19.5)	501 (16.2)	8.6
gMG symptoms				
Ptosis	2,366 (38.2)	1,047 (39.4)	1,121 (36.3)	6.5
Diplopia	2,357 (38.0)	999 (37.6)	1,169 (37.8)	0.5
General and other muscle-specific weaknesses	1,956 (31.6)	1,063 (40.0)	910 (29.4)	22.3^*^
Dyspnea	1,751 (28.3)	932 (35.1)	798 (25.8)	20.3^*^
Malaise and fatigue	1,730 (27.9)	840 (31.6)	833 (26.9)	10.3^*^
Dysphagia	1,350 (21.8)	785 (29.5)	519 (16.8)	30.6^*^
Dysarthria	503 (8.1)	312 (11.7)	163 (5.3)	23.4^*^
**All-cause pharmacy and medical costs, PPPY**	27,737 ± 49,157 [12,373]	35,529 ± 64,585 [16,754]	26,141 ± 45,284 [10,571]	16.8^*^
Medical costs	22,545 ± 41,328 [9,323]	26,794 ± 50,256 [12,201]	20,654 ± 38,037 [7,942]	13.8^*^
Pharmacy costs	5,192 ± 21,337 [707]	8,735 ± 37,020 [1,099]	5,487 ± 20,065 [739]	10.9^*^

DSM-5, Diagnostic and Statistical Manual of Mental Disorders, 5th Edition; gMG, generalized myasthenia gravis; PPPY, per-patient-per-year; SD, standard deviation; Std. diff, standardized difference. ^*^indicates std. diff >10%. ^a^Std. diff. was calculated between the *clinical event* and *no clinical event* cohorts.

During the follow-up period (mean duration: 33.4 months), 3,045 patients had a clinical event (49.2%), of which, 98.2% were MEs and 1.8% were MCs. The first clinical event occurred, on average, 5.4 months after the index date.

Male patients had 15% higher likelihood of experiencing a clinical event compared to females (HR: 1.15; *p* = 0.017; Figure 1). Baseline gMG symptoms were also associated with higher likelihood: dysarthria increased the likelihood by 45% (1.45; *p* < 0.001), generalized or other weaknesses by 38% (1.38; *p* < 0.001), dysphagia by 28% (1.28; *p* < 0.001), dyspnea by 23% (1.23; *p* = 0.001), and ptosis by 20% (1.20; *p* = 0.001). A shorter time from first MG diagnosis to the first evidence of gMG also increased the likelihood, with each fewer month increasing the likelihood by 6% (1.06; *p* < 0.001). The use of immunoglobulin and non-steroidal immunosuppressants increased the likelihood by 54% (1.54; *p* < 0.001) and 37% (1.37; *p* = 0.026), respectively. Weight loss increased the likelihood by 27% (1.27; *p* = 0.009), while the absences of other neurological conditions or autoimmune disorders increased the likelihood by 20% (1.20; *p* = 0.007) and 18% (1.18; *p* = 0.040), respectively. Additionally, each additional 10 ED visits during baseline increased the likelihood by 30% (1.30; *p* < 0.001).

**Figure 1:**
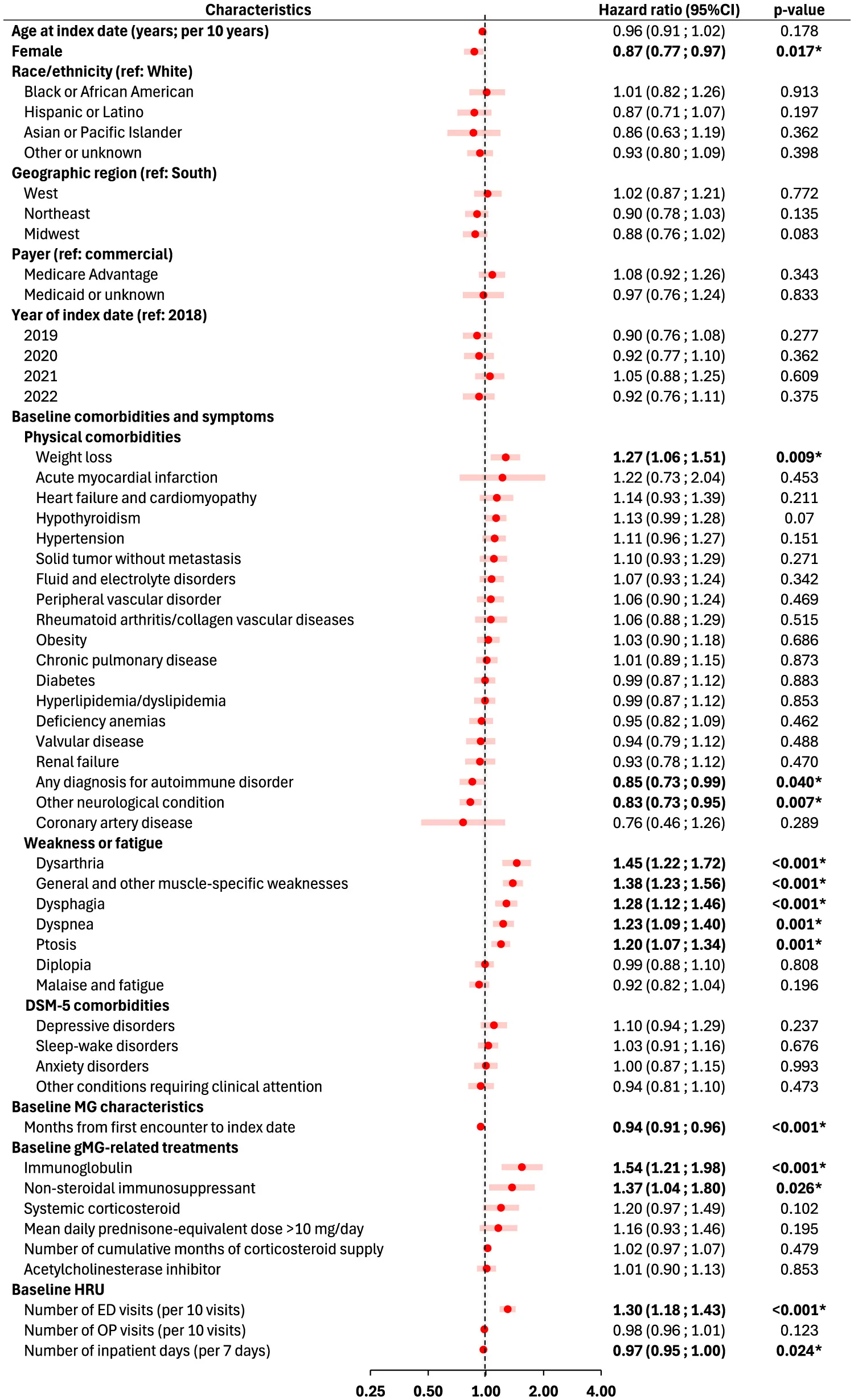
Predictors of a clinical event. CI, confidence interval; DSM-5, Diagnostic and Statistical Manual of Mental Disorders, 5th Edition; ED, emergency department; gMG, generalized myasthenia gravis; HRU, healthcare resource utilization; MG, myasthenia gravis; OP, outpatient. *indicates *p*-value <0.05.

### 3.2 Healthcare resource utilization

After the application of selection criteria and entropy balancing, 2,657 patients were included in the *clinical event* cohort, and 3,092 patients were included in the *no clinical event* cohort. The weighted baseline characteristics of both cohorts were similar to those of the *gMG* cohort and well balanced. The mean age at index was 61.4 years, 47.5% were female, 64.0% were White, and 52.4% had commercial insurance coverage (Table 1).

The most common physical comorbidities for both cohorts were hypertension (*clinical event* cohort: 62.0%; *no clinical event* cohort: 58.7%), obesity (37.0% and 34.1%), and diabetes (27.7% and 25.8%, respectively).

During the follow-up period, and for all-cause events, patients in the *clinical event* cohort had 5.82 times the number of inpatient days (*clinical event* cohort: 9.01 days; *no clinical event* cohort: 1.55 days; *p* < 0.001), 6.02 times the number of inpatient admissions (0.91 and 0.15; *p* < 0.001), 1.91 times the number of ED visits (1.44 and 0.76; *p* < 0.001), and 1.58 times the number of outpatient visits (41.27 and 26.04; *p* < 0.001) compared to those in the *no clinical event* cohort (Figure 2). Further, for gMG-related events, patients in the *clinical event cohort* had 16.09 times the number of gMG-related inpatient admissions (0.82 and 0.05; *p* < 0.001), 12.23 times the number of gMG-related ED visits (0.52 and 0.04; *p* < 0.001), and 3.96 times the number of gMG-related outpatient visits (18.29 and 4.62; *p* < 0.001) compared to patients in the *no clinical event* cohort.

**Figure 2:**
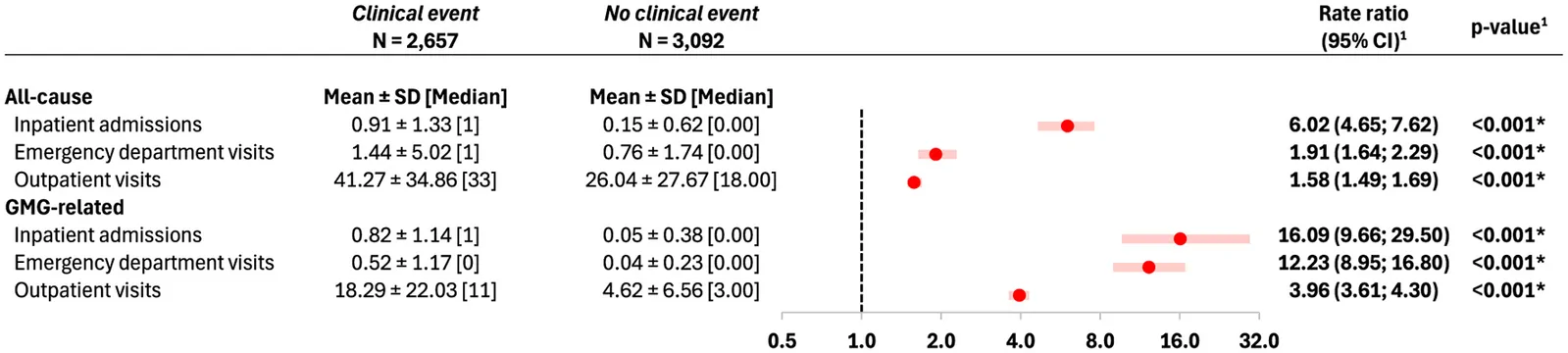
Healthcare resource utilization among patients with and without a clinical event (PPPY) during the follow-up period. CI, confidence interval; gMG, generalized myasthenia gravis; PPPY, per-patient-per-year; SD, standard deviation. *indicates *p*-value <0.05. [1] Rate ratios were estimated using weighted Poisson regression models, and 95% CIs and *p*-values were estimated using a non-parametric bootstrapping procedure (r = 500). Rate ratios >1 indicate that the *clinical event cohort* had a higher rate of having the outcome compared to the *no clinical event* cohort.

### 3.3 Healthcare costs

During the baseline period, the mean all-cause total healthcare costs PPPY were $35,529 for the *clinical event* cohort and $26,141 for the *no clinical event* cohort.

During the follow-up period, the mean all-cause total healthcare costs PPPY among the *clinical event* cohort were nearly 4 times as high as those in the *no clinical event* cohort (*clinical event* cohort: $95,821; *no clinical event* cohort: $25,029; MCD: $70,792; *p* < 0.001; Figure 3A). This cost difference was driven almost equally by pharmacy costs and medical costs, which respectively accounted for $35,270 and $35,522 of the total cost difference. The difference in immunoglobulin costs accounted for $18,103, corresponding to 51.3% of the difference in pharmacy costs between the *clinical event* and *no clinical event* cohorts. The difference in medical costs was driven mainly by inpatient costs (*clinical event* cohort: $29,039; *no clinical event* cohort: $3,768; MCD: $25,271; *p* < 0.001), accounting for 71.1% of the total medical cost difference between the cohorts.

**Figure 3:**
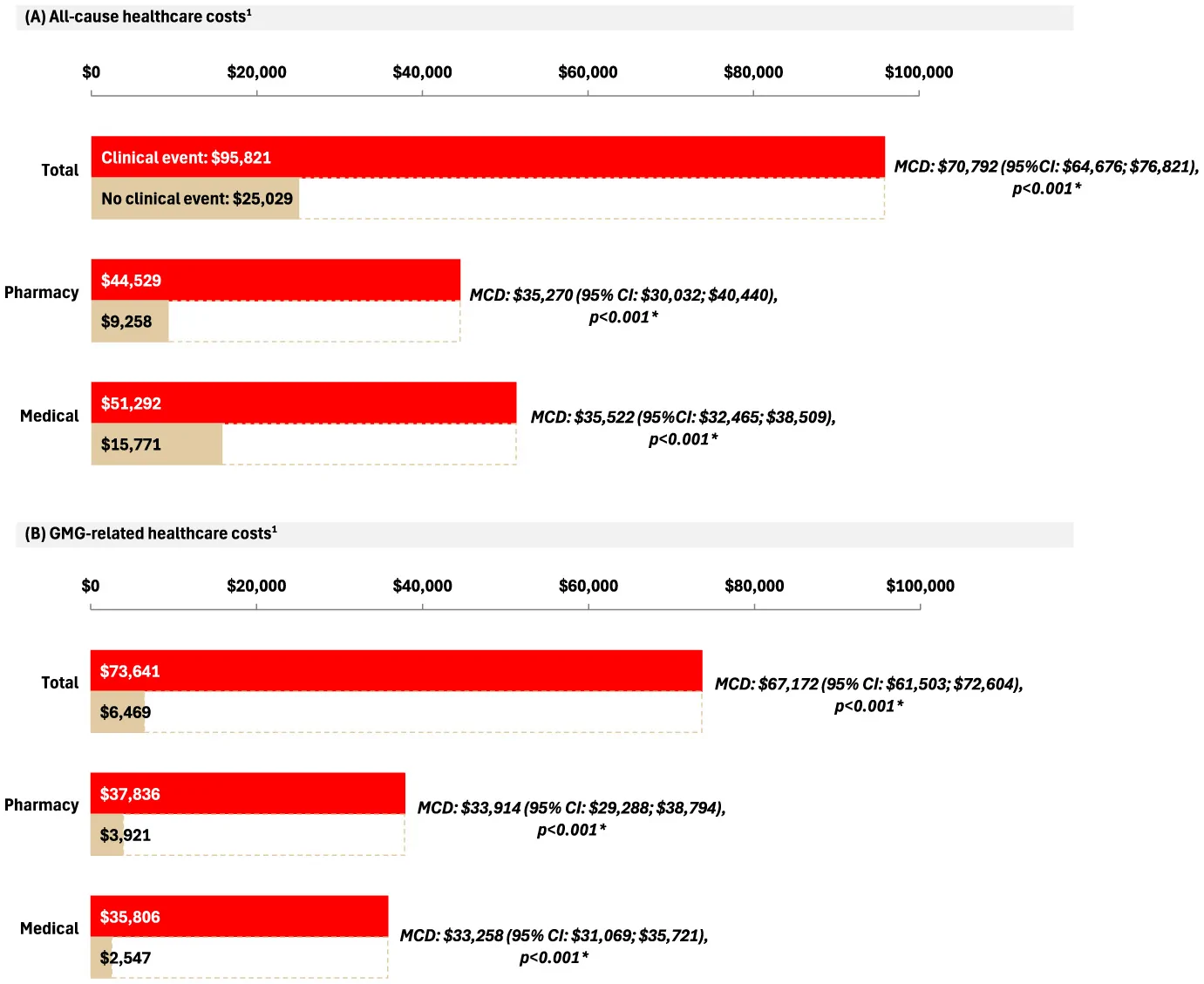
Healthcare costs PPPY (2023 USD) among patients with and without a clinical event during the follow-up period. **(A)** All-cause healthcare costs^1^. **(B)** GMG-related healthcare costs^1^. CI, confidence interval; gMG, generalized myasthenia gravis; MCD, mean cost difference; PPPY, per-patient-per-year; USD, United States dollars. *indicates *p*-value <0.05. [1] MCDs were estimated using weighted ordinary least squares regression models, and 95% CIs and *p*-values were estimated using a non-parametric bootstrapping procedure (r = 500).

The mean gMG total healthcare costs PPPY during the follow-up period among the *clinical event* cohort were over 11 times as high as those in the *no clinical event* cohort (*clinical event* cohort: $73,641; *no clinical event* cohort: $6,469; MCD: $67,172; *p* < 0.001; Figure 3B). Similar to the all-cause healthcare costs, the difference in gMG-related costs was driven almost equally by pharmacy costs ($33,914) and medical costs ($33,258).

Among patients in the *clinical event* cohort, the mean monthly all-cause healthcare costs were $5,786 during the month before the first clinical event and increased to $19,325 during the month following the clinical event (Figure 4). The costs then declined, but remained high in subsequent months, ranging from $6,626 to $7,268 each month.

**Figure 4:**
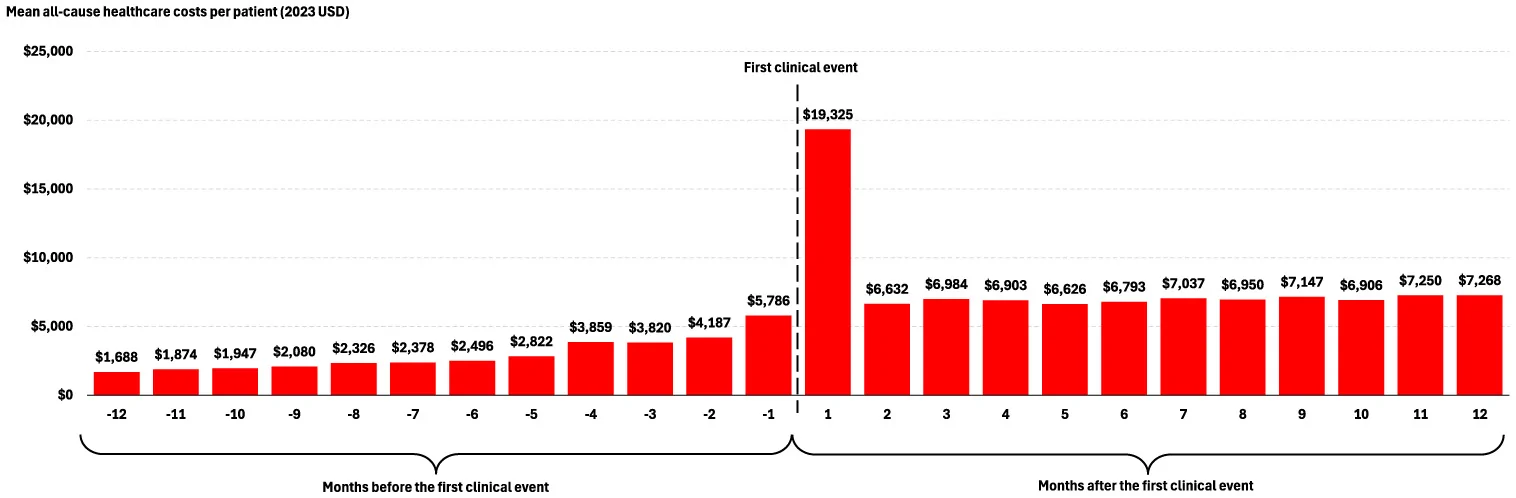
Total all-cause healthcare cost distribution before and after the first clinical event. USD, United States dollars.

## 4 Discussion

In this real-world study of adults with newly diagnosed gMG, key predictors of the first ME or MC event included male sex, greater disease severity as indicated by weakness symptoms, the need for acute rescue therapies before the evidence of gMG, and the time of progression from first MG diagnosis to first gMG confirmation by a neurologist. The first ME or MC event was associated with a substantial economic burden, reflected in increased HRU and healthcare costs. Pharmacy costs, particularly those related to immunoglobulin, accounted for approximately half of the incremental cost burden, while the remaining half was attributable to medical costs, primarily from inpatient hospitalizations. Considered together, these findings suggest that patients presenting with these risk factors were not only more likely to experience a clinical event, but also to incur substantially greater downstream HRU and costs, underscoring the value of early identification.

This analysis identified several important predictors associated with the risk of MEs and MCs in patients with gMG. Specific gMG symptoms, including dysarthria, dysphagia, dyspnea, ptosis, and muscle-specific weaknesses being present before a neurologist-confirmed evidence of gMG were associated with higher risk MEs or MCs, highlighting the need for more efficacious treatment to prevent or control symptoms before they lead to a clinical event. A more rapidly progressing course, as proxied by a shorter interval between first MG diagnosis and the initial neurologist confirmation of gMG, as well as the use of rescue therapy during baseline, also increased the risk. Early neurologist involvement and rescue therapy may also serve as an indicator of disease severity, underscoring the need for early risk stratification, close monitoring, and timely treatment initiation. Prior studies have shown that progressive respiratory weakness, including dysphagia, dysarthria, and dyspnea, is a key warning sign of imminent MC and often precedes ventilatory failure (9, 23, 24). Our findings are also consistent with studies identifying these symptoms as independent risk factors for MEs (18, 25).

Interestingly, patients with other autoimmune or neurological disorders had lower risk of clinical events, which may be related to receiving more intensive clinical monitoring or receiving newer and more advanced medications. For example, more intensive follow-up or monitoring was associated with better status and fewer severe deteriorations in patients with MG in a large multicenter cohort study; patients who achieved minimal manifestation status by 12 months also had lower subsequent risk of ME or MC (18). Additionally, newer advanced therapies have been shown to reduce MG worsening and the need for rescue therapies in randomized and observational studies (26, 27); patients already receiving such treatments for another autoimmune condition could potentially experience fewer MG events. The use of immunoglobulin or non-steroidal immunosuppressants (e.g., azathioprine, mycophenolate, cyclosporine) during baseline were both associated with higher risk of clinical events, which may be because patients with severe disease are more likely to receive these therapies per clinical guidelines (28, 29).

A substantial portion of the economic toll associated with MEs and MCs stems from high pharmacy costs, particularly those related to immunoglobulin therapy. This aligns with recent real-world data demonstrating that patients with MG in Norway treated with chronic intravenous immunoglobulin incur more than double the healthcare costs compared with patients not receiving it in the first year post-diagnosis, with costs remaining elevated through 5 years of follow-up (30). Another analysis of US patients with gMG estimated that immunoglobulin costs composed over half of all medical costs for intermittent immunoglobulin users and over 80% in chronic immunoglobulin users (31). Given that immunoglobulin preparations confer only temporary relief, with clinical benefits lasting only 4–5 weeks, their recurrent use highlights a pressing unmet need for more durable therapeutic strategies (32, 33).

MEs and MCs in gMG necessitate substantial additional medical care, reflected in both inpatient and outpatient settings. The difference in inpatient costs accounted for over a third of the total economic burden, as MEs often require treatment in the ED or inpatient setting, with rescue therapies such as intravenous immunoglobulin administered alongside close monitoring and surveillance. MCs are particularly resource-intensive, necessitating inpatient admission and frequent intubation for respiratory support. Beyond direct gMG management, MEs and MCs also increase HRU through complications not specific to MG. For example, intensive immunosuppression and treatment-related adverse effects predispose patients to infections (9).

Importantly, the economic impact of MEs and MCs is not limited to the acute event period. Compared to the period before the clinical event, healthcare costs increased sharply at the time of ME or MC onset and remained substantially elevated during the subsequent year, reflecting ongoing management (16). This sustained cost burden underscores that MEs and MCs represent not only transient clinical events, but also prolonged periods of intensified healthcare utilization with lasting economic consequences.

Beyond the comorbidities examined in this study, non-motor manifestations and systemic complications, such as myocarditis, may represent additional aspects of gMG-related burden that were not captured in our analyses; future research incorporating a broader range of these manifestations may help further characterize the overall burden of gMG (34).

A key strength of this study is that it reflects the relatively contemporary treatment landscape for gMG, capturing data through 2023 and thereby encompassing the impact of recently approved therapies and evolving patterns of care. The large size of each analytic cohort provided sufficient statistical power to generate reliable and generalizable findings. In addition, the use of KRD+ data offered the advantage of available provider specialty for each medical encounter, allowing confirmation of neurologist involvement in the diagnosis and management of gMG. This approach strengthened the analysis by establishing a clear start of follow-up at the point of a neurologist-confirmed gMG event. Taken together, these strengths help support the validity of the results, which underscore an ongoing unmet need for newer and more advanced therapies that can achieve better symptom control and delay disease progression to ME or MC.

The results of this study should be considered in the light of several limitations, some of which are inherent to analyses of administrative claims data such as miscoding or misclassification. First, because no specific ICD-10-CM diagnostic code exists for gMG, patient identification relied on a claims-based algorithm that incorporated MG diagnoses, care settings, and physician specialty. This algorithm, originally proposed by Phillips et al. and subsequently refined in the literature as well as in the present study (19, 20), provides a pragmatic but indirect approach. Relatedly, the ICD-10-CM codes used to identify MG (G70.00, G70.01) do not distinguish generalized from purely ocular MG. Although requiring a neurologist diagnosis likely enriched the cohort for gMG, inclusion of some patients with ocular MG remains possible in the absence of clinical confirmation. Additionally, the index date reflected the first neurologist-confirmed evidence of gMG in claims data and was used as a proxy for the start of gMG; given the nature of disease progression, some patients may have had earlier MG-related diagnoses, symptoms, or treatments, some of which may not have been MG-specific. Second, MEs and MCs were identified using diagnosis and procedure codes, and misclassification could not be excluded, although several criteria (e.g., requiring a distinct encounter, and for MC, ventilatory support coded on the same claim as the MG diagnosis) were applied to improve specificity. Further, claims data did not capture standardized clinical severity measures (e.g., MG-Activities of Daily Living scale, MG Composite score, MGFA Severity Classifications) that would allow more granular characterization of event severity. Third, clinical events were identified inclusively, without excluding encounters involving concurrent precipitating conditions (e.g., infections), and HRU and costs associated with managing such conditions during a clinical event were considered part of its overall real-world burden rather than removed as confounding; nonetheless, residual confounding also remains possible, given the absence of information on unmeasured factors such as lifestyle, socioeconomic status, and social support. Fourth, the study period overlapped with the COVID-19 pandemic, which may have influenced patterns of care and utilization. Finally, the findings may not be generalizable to individuals without health insurance or those covered by payers other than commercial plans, Medicare Advantage, or Medicaid. Together with the observational and descriptive nature of claims data, these limitations prevent causal inference.

## 5 Conclusion

This real-world analysis shows that nearly half of patients with gMG experienced an ME or MC. Greater disease severity and use of immunosuppressive therapy at baseline were associated with a higher risk of clinical events, which in turn were linked to substantial HRU and costs. Collectively, these findings may help identify patients who could benefit from more advanced gMG treatments and underscore an unmet need for more effective therapies to improve symptom control and reduce the risk of acute clinical events.

## Data Availability

The datasets presented in this article are not readily available because the data that support the findings of this study are available from Komodo^®^. Restrictions apply to the availability of these data, which were used under license for this study. Requests to access the datasets should be directed to https://www.komodohealth.com/.
